# Drug-induced risk of depression: A 20-year real-world pharmacovigilance analysis based on the FAERS database

**DOI:** 10.1097/MD.0000000000047076

**Published:** 2026-01-09

**Authors:** Xinping Xiang, Li Yin, Chunyan Deng, Qiuxia Feng

**Affiliations:** a Child and Adolescent Psychology Center, Nanchong Mental Health Center of Sichuan Province, Nanchong, Sichuan Province, China; b Quality Management Department, Nanchong Mental Health Center of Sichuan Province, Nanchong, Sichuan Province, China; c Outpatient Department, Nanchong Psychosomatic Hospital, Nanchong, Sichuan Province, China.

**Keywords:** adverse drug events, data mining, depression, disproportionality analysis, signal detection

## Abstract

With the advancement of global healthcare and drug research, drug-induced adverse reactions, particularly drug-induced adverse effects of depression (DIAEs), are garnering increased attention. DIAEs significantly impact patients’ quality of life, treatment adherence, and prognosis, potentially exacerbating conditions, leading to medication discontinuation, or triggering extreme events like suicide. Therefore, exploring DIAEs mechanisms and effects is crucial. The aim of this study was to comprehensively explore and analyze drug-induced depressive adverse events (DIAEs) using the US Food and Drug Administration’s (FDA’s) Adverse Event Reporting System (FAERS) database, which provides a scientific basis for drug safety monitoring, clinical medication guidance, and improvement of drug development. In this study, we analyzed in-depth the time of onset, drug class, drug interactions, and demographic characteristics of DIAEs by data cleaning and preprocessing the reports of depression-related adverse events (AEs) in the FAERS database from the first quarter of 2004 to the third quarter of 2024, using statistical methods and data mining techniques. The disproportionate analysis (DPA) algorithm was used to combine multiple algorithms (e.g., ROR, PRR, BCPNN, EBGM) for signal detection of DIAEs-related drugs. It was found that the number of depression-related drug reports in the FAERS database increased year by year, and the trend of depressive AEs showed a polynomial growth curve with high *R*^2^ values. The analysis showed that drugs such as Varenicline, Isotretinoin and Adalimumab were highly associated with depressive AEs and that the risk associated with depression was not mentioned in the labeling of 12 drugs, revealing new drug signals. Analysis of the time to onset of DIAEs revealed an “early failure curve” for many drugs, with a median time to onset of depression of 27 days for Varenicline. The in-depth analysis of DIAEs in the FAERS database revealed the epidemiological characteristics, demographic distribution, and potential risk factors of depressive AEs, which provides an important basis for drug safety monitoring, clinical decision-making, and drug labeling updates. The study also identified multiple signals of drug depression that were not labeled in drug labels, suggesting that the monitoring of high-risk drugs should be strengthened in clinical practice.

## 1. Introduction

With advancements in global healthcare and rapid development in drug research and development,^[1]^ the potential adverse drug reactions (ADRs) associated with therapeutic medications have increasingly garnered attention.^[2]^ Among these, drug-induced depressive adverse events (DIAEs) stand out as a severe mental health issue, profoundly impacting patients’ quality of life, treatment adherence, and prognosis.^[3,4]^ DIAEs can exacerbate existing conditions, lead to treatment discontinuation,^[5]^ and even trigger extreme events such as suicide, highlighting the importance of their in-depth exploration and analysis.^[6,7]^

The Food and Drug Administration Adverse Event Reporting System (FAERS), maintained by the United States Food and Drug Administration (FDA), is one of the largest public drug safety databases worldwide. It harbors vast real-world data sourced from patients, physicians, and pharmaceutical companies, providing invaluable resources for drug safety research. The FAERS database not only encompasses detailed information on ADRs but also covers multidimensional data such as patient age, gender, body weight, past medical history, comorbidities, and medication history, enabling a comprehensive assessment of DIAEs.^[8]^

Although recent years have witnessed several studies on DIAEs, they have largely focused on specific drugs or populations, lacking comprehensiveness and systematicity.^[9]^ Furthermore, in-depth analysis of the onset time, demographic characteristics, drug classes, and drug interactions associated with DIAEs remains insufficient.^[10-12]^ Notably, various drugs have been reported to cause depressive adverse reactions in clinical practice.^[13]^ For instance, antidepressant medications themselves, such as fluoxetine, sertraline, and paroxetine within selective serotonin reuptake inhibitors (SSRIs),^[14]^ and venlafaxine and Duloxetine among serotonin-norepinephrine reuptake inhibitors (SNRIs), may cause adverse reactions like nausea, vomiting, sexual dysfunction, and worsening depressive symptoms post-administration.^[15-17]^ Additionally, antipsychotics like chlorpromazine, olanzapine, and risperidone can induce depressive symptoms in patients when treating schizophrenia, bipolar disorder, and other conditions.^[18-21]^ Certain antihypertensive drugs, such as the β-blocker propranolol and certain diuretics, as well as opioid analgesics like morphine and meperidine, may also trigger or exacerbate depressive symptoms when used chronically or at high doses.^[22-25]^ However, the specific mechanisms underlying drug-induced depression remain incompletely understood, potentially involving drug effects on neurotransmitters, individual genetic backgrounds, and environmental factors.^[5]^

Therefore, this study aims to comprehensively mine and analyze DIAEs using real-world data from the FAERS database, aiming to provide a scientific basis for drug safety monitoring, clinical medication guidance, drug regulatory decision-making, and improvements in drug research and development. Specifically, we will first screen depression-related AE reports through data cleaning and preprocessing. Subsequently, we will employ statistical methods to deeply analyze key information on the onset time, demographic characteristics (e.g., age, gender, body weight), drug classes, and drug interactions associated with DIAEs. Furthermore, this study will utilize data mining techniques to identify drugs most closely associated with DIAEs and explore their possible mechanisms. Through these analyses, we hope to reveal the underlying patterns of DIAEs, providing more refined and scientific guidance for clinical practice and drug research and development.

In summary, this study will delve into the mining and analysis of DIAEs based on real-world data from the FAERS database, aiming to offer new perspectives and methodologies for research in the field of drug safety, while also providing safer and more effective treatment options for patients.

## 2. Materials and methods

### 2.1. Data sources and process

The pharmacovigilance data analyzed in this study were extracted from the Food and Drug Administration Adverse Event Reporting System (FAERS; https://fis.fda.gov/extensions/FPD-QDE-FAERS/FPD-QDE-FAERS.html), a publicly accessible post-marketing surveillance repository established in 2004. Our analysis encompassed reports spanning from the first quarter of 2004 through the third quarter of 2024, retrieved in ASCII format from the FAERS platform.

To ensure data integrity, duplicate entries sharing identical “caseid” identifiers were systematically removed, retaining only the most recent submission based on the event documentation date. Drug nomenclature was harmonized through alignment with the RxNorm standardized medication classification system, resolving inconsistencies in pharmaceutical product designations across FAERS records. AE terminology standardization was achieved by mapping reported terms to the Medical Dictionary for Regulatory Activities (MedDRA v27.0) preferred terms (PTs), with specific focus on depression-related outcomes.

Following data normalization procedures, we specifically identified AE reports where depression was documented as a primary outcome in conjunction with medications classified as primary suspect (PS) agents. The curated dataset was subsequently characterized through multidimensional analysis of demographic variables (gender, age, weight), clinical parameters (therapeutic indications), geographic distribution (reporting countries), and patient outcomes.

### 2.2. Signal analysis algorithm

This study employed disproportionality analysis (DPA), a quantitative pharmacovigilance methodology, to identify potential drug-associated depression signals. The DPA framework utilizes 2 × 2 contingency tables (Table 1) to quantify drug-AE correlations through comparative frequency analysis between medication-exposed and nonexposed populations. Four complementary disproportionality metrics were implemented for signal quantification: reporting odds ratio (ROR), proportional reporting ratio (PRR), Bayesian Confidence Propagation Neural Network (BCPNN), Multi-item Gamma Poisson Shrinker (MGPS). Signal validity required concurrent satisfaction of all 4 algorithms’ positivity thresholds (Table 2). Post-identification verification involved cross-referencing detected signals against FDA-approved drug labeling documents (accessed via https://www.accessdata.fda.gov/scripts/cder/daf/index.cfm), with novelty designation assigned to signals absent from official prescribing information.

**Table 1:** Two-by-two contingency table for disproportionality analyses.

Drugs	Target AEs	Other AEs	Total
Target drugs	*a*	*b*	*a* + *b*
Other drugs	*c*	*d*	*c* + *d*
Total	*a* + *c*	*b* + *d*	*a* + *b* + *c* + *d*

*a*, number of reports containing both the target drug and target adverse drug reaction; *b*, number of reports containing other adverse drug reaction of the target drug; *c*, number of reports containing the target adverse drug reaction of other drugs; and *d*, number of reports containing other drugs and other adverse drug reactions.

AE = adverse event.

**Table 2:** Four major algorithms used for signal detection.

Algorithms	Equation	Criteria
ROR	ROR = *ad*/*b*/*c*	Lower limit of 95% CI > 1, N ≥ 3
	95% CI = e^ln(ROR)±1.96(1/*a*+1/*b*+1/*c*+1/*d*)^0.5^	
PRR	PRR = *a*(*c* + *d*)/*c*/(*a* + *b*)	PRR ≥ 2, χ^2^ ≥ 4, N ≥ 3
	χ^2^ = [(*ad* – *bc*)^2^](*a* + *b* + *c* + *d*)/[(*a* + *b*)(*c* + *d*)(*a* + *c*)(*b* + *d*)]	
BCPNN	IC = log_2_*a*(*a* + *b* + *c* + *d*)(*a* + *c*)(*a* + *b*)	IC025 > 0
	95% CI = E(IC) ± 2V(IC)^0.5^	
MGPS	EBGM = *a*(*a* + *b* + *c* + *d*)/(*a* + *c*)/(*a* + *b*)	EBGM05 > 2
	95% CI = e^ln(EBGM)±1.96(1/*a*+1/*b*+1/*c*+1/*d*)^0.5^	

*a*, number of reports containing both the target drug and target adverse drug reaction; *b*, number of reports containing other adverse drug reaction of the target drug; *c*, number of reports containing the target adverse drug reaction of other drugs; and *d*, number of reports containing other drugs and other adverse drug reactions.

χ^2^ = chi-squared, BCPNN = Bayesian Confidence Propagation Neural Network, CI = confidence interval, E(IC) = the IC expectations, MGPS = Multi-item Gamma Poisson Shrinker, EBGM = empirical Bayesian geometric mean, EBGM05 = the lower limit of 95% CI of EBGM, IC = information component, IC025 = the lower limit of 95% CI of the IC, V(IC) = the variance of IC, N = the number of reports, PRR = proportional reporting ratio, ROR = reporting odds ratio.

**Figure 1.**
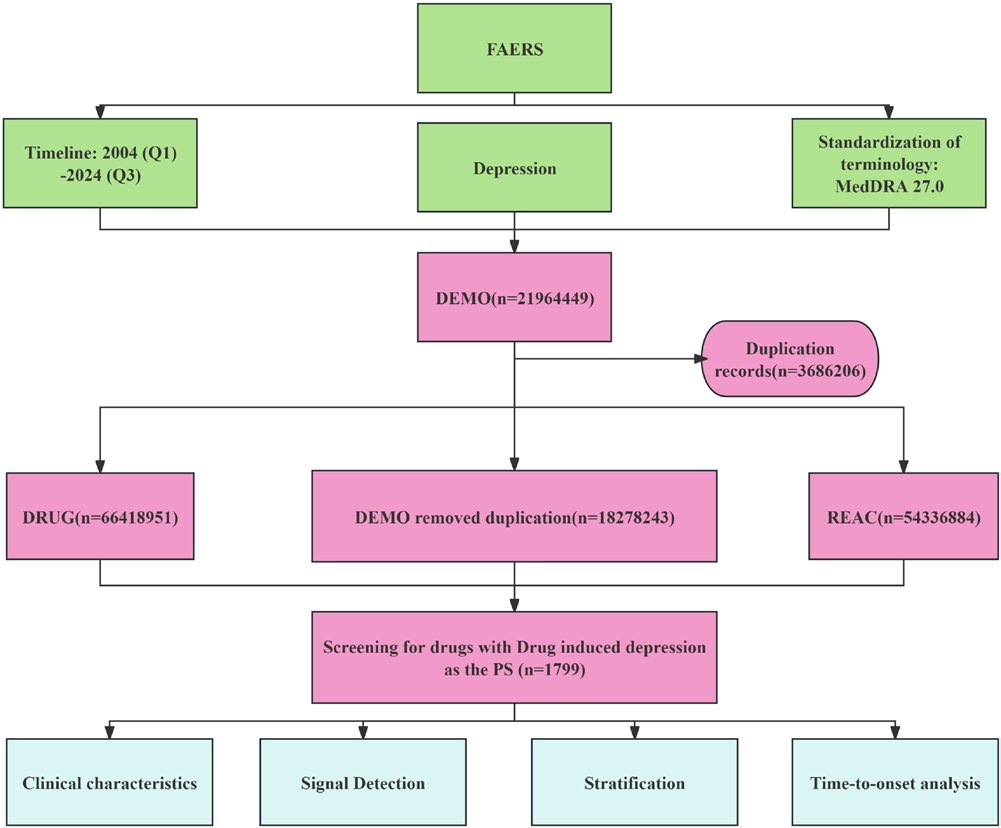
Analysis workflow for drug-induced depressive events. FAERS = Food and Drug Administration Adverse Event Reporting System, MedDRA = Medical Dictionary for Regulatory Activities.

All analytical workflows, including data processing and statistical evaluation, were executed using R v4.4.0 for computational analysis and Microsoft Excel for supplementary data management. The integrated methodological pipeline is schematically presented in Figure 1.

## 3. Results

### 3.1. Basic characteristics of adverse events related to depression

**Figure 2.**
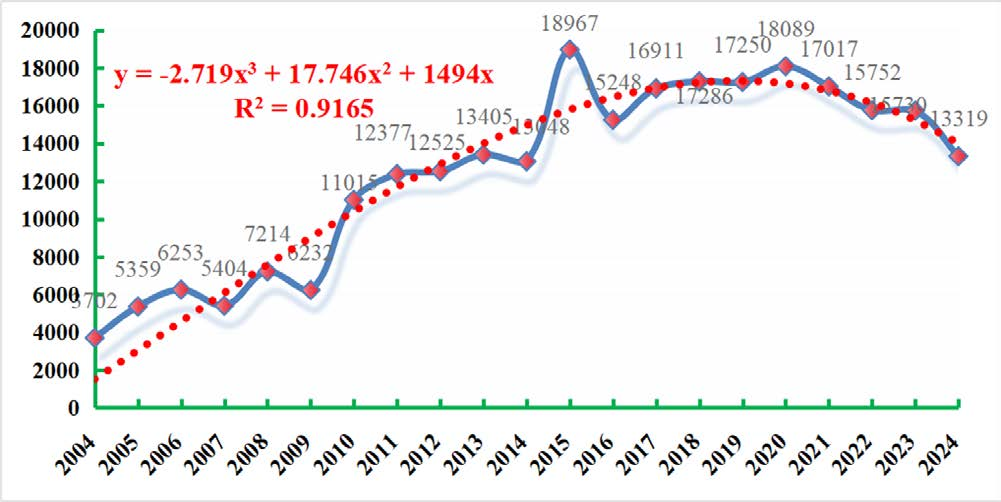
Trend in reporting of drug-induced depressive adverse events.

As of the third quarter of 2024, the number of AE reports related to depression in the FAERS database was 2,62,103. From the first quarter of 2004 to the third quarter of 2024, the number of AE reports with “depression” as the preferred term increased annually, peaking at 18,967 in 2015 (see Fig. 2). The polynomial fitting curve exhibits a trend of gradual growth, with a coefficient of determination (*R*^2^ = 0.9165) indicating that the model explains 91.65% of the data variability. This trend has high reference value. Table 3 displays the characteristics of the population experiencing depressive AEs, among which 61.2% were female, 32.2% were male, and 6.6% had unknown gender. In terms of age stratification, there is a gradual increase, with the 18 to 65 age group accounting for the largest proportion (47.77%) of AEs with known age. Depressive AEs were most common in individuals weighing 70 to 90 kg (15.69%), but 65.09% of the population had unknown weight. Notably, multiple sclerosis (7.0%) had the highest incidence of depression as an indication, followed by depression (4.3%) and rheumatoid arthritis (3.5%). Depressive AEs were mainly concentrated in developed countries, with the United States accounting for 67.09%, the United Kingdom for 6.33%, and Canada for 4.98%.

**Table 3:** Baseline characteristics of the drug-induced depression population.

Characteristics	Case numbers	Case proportion (%)
Number of events	262,103	–
Gender		
Female	160,452	61.2%
Male	84,456	32.2%
Miss	17,195	6.6%
Age		
Median age	50	
<18	9,419	3.59%
18–65	125,198	47.77%
65–85	30,261	11.55%
>85	2,891	1.10%
Miss	94,334	35.99%
Weight (kg)		
<50	2,603	0.99%
50–69	16,553	6.32%
70–89	41,136	15.69%
≥90	31,215	11.91%
Miss	170,596	65.09%
Top 5 indication		
Multiple sclerosis	18,438	7.0%
Depression	11,210	4.3%
Rheumatoid arthritis	9,061	3.5%
Smoking cessation therapy	8,041	3.1%
Contraception	6,627	2.5%
Top 5 reported countries		
United States	175,848	67.09%
United Kingdom	16,600	6.33%
Canada	13,049	4.98%
France	7,213	2.75%
Brazil	4,156	1.59%

### 3.2. Drug analysis

**Figure 3.**
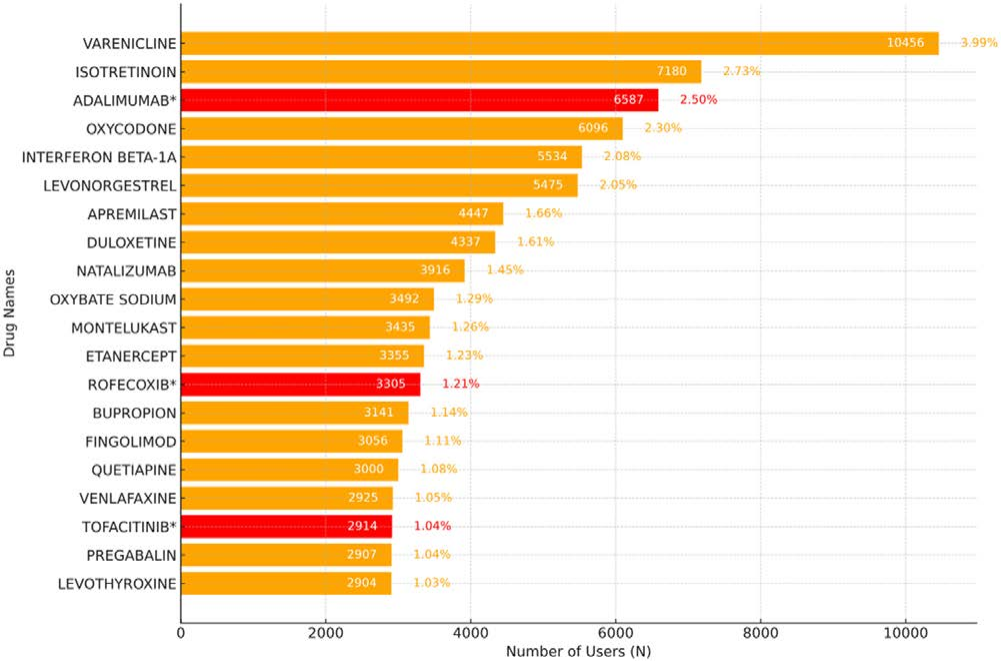
Top 20 drugs with the highest number of reports related to drug-induced depression. * indicates a new signal not mentioned in the prescribing information.

The top 20 drugs associated with the highest number of drug-induced depressive AEs are shown in Figure 3. The top 5 drugs are Varenicline (with 10,456 reports), Isotretinoin (7180 reports), Adalimumab (6587 reports), Oxycodone (6096 reports), and Interferon beta-1A (5534 reports). Notably, Adalimumab’s prescribing information does not explicitly mention the risk of drug-induced depressive AEs. Among the top 20 drugs with the highest number of drug-induced depressive AEs, 3 drugs have labeling that does not mention the risk of depressive events.

### 3.3. Signal detection of adverse events related to drug-induced depression

The statistical results of the disproportionality analysis indicate that among the top 20 drugs with the strongest signals of AE intensity, the labeling of 12 drugs does not mention the risk of drug-induced depression, representing a new AE signal. The top 4 drugs in terms of the strength of this new signal are ESTRONE [n = 2, ROR 68.74 (9.68–487.99), PRR 34.87 (66.75), EBGM 34.87 (6.76), IC 5.12 (3.02)], ASCORBIC ACID* [n = 1, ROR 68.74 (4.3–1098.99), PRR 34.87 (33.38), EBGM 34.87 (3.43), IC 5.12 (2.49)], BETIBEGLOGENE AUTOTEMCEL* [n = 1, ROR 68.74 (4.3–1098.99), PRR 34.87 (33.38), EBGM 34.87 (3.43), IC 5.12 (2.49)], ETYNODIOL* [n = 1, ROR 68.74 (4.3–1098.99), PRR 34.87 (33.38), EBGM 34.87 (3.43), IC 5.12 (2.49)], INDORAMIN [n = 1, ROR 68.74 (4.3–1098.99), PRR 34.87 (33.38), EBGM 34.87 (3.43), IC 5.12 (2.49)].

**Figure 4.**
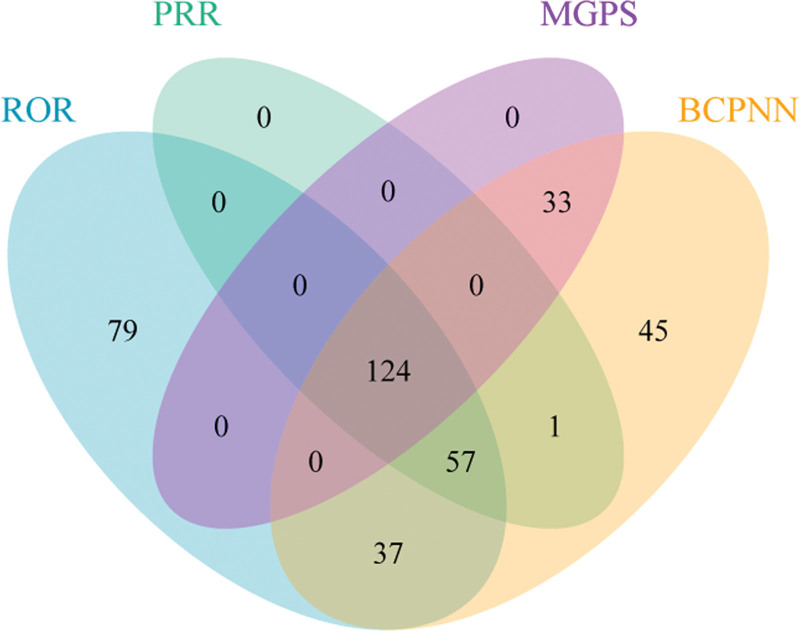
The Venn diagram shows the number of drugs that show positive results in all 4 algorithms. BCPNN = Bayesian Confidence Propagation Neural Network, MGPS = Multi-item Gamma Poisson Shrinker, PRR = proportional reporting ratio, ROR = reporting odds ratio.

**Figure 5.**
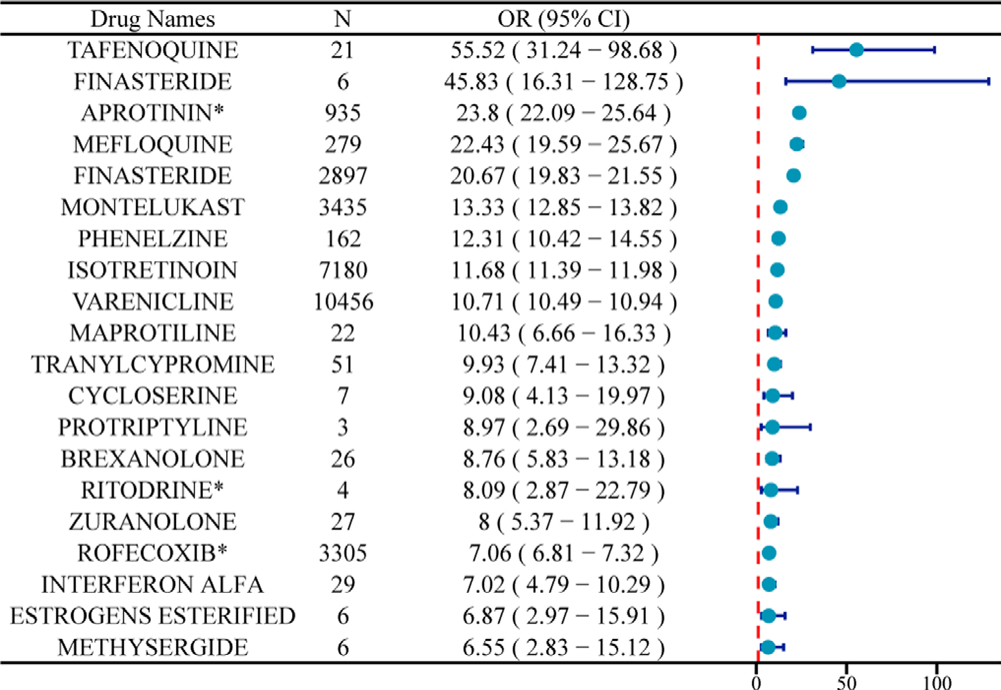
The forest map is used to show drugs that are positive in all 4 algorithms and have strong signal strength. CI = confidence interval, OR = odds ratio.

The drug with the highest number of reports and no mention of this AE in its labeling is APROTININ* [n = 935, ROR 23.8 (22.09–25.64), PRR 17.95 (15,128.02), EBGM 17.89 (16.81), IC 4.16 (4.06)]. Figure 4 presents a Venn diagram of the 4 algorithms: ROR, PRR, MGPS, and BCPNN, with 124 drugs showing positive results across all 4 algorithms. To further identify more rigorous adverse reaction events, we plotted a forest plot (Fig. 5) for drugs that were positive across all 4 algorithms and had strong signal intensities. Among these drugs, the prescribing information for 3 drugs – APROTININ, RITODRINE, and ROFECOXIB* – did not explicitly mention AEs related to drug-induced depression (Table 4).

**Table 4:** Top 20 drugs ranked by signal strength.

Drug names	N	ROR (95% CI)	PRR (X^2^)	EBGM (EBGM05)	IC (IC025)
Estrone*	2	68.74 (9.68–487.99)	34.87 (66.75)	34.87 (6.76)	5.12 (3.02)
Ascorbic acid*	1	68.74 (4.3–1098.99)	34.87 (33.38)	34.87 (3.43)	5.12 (2.49)
Betibeglogene autotemcel*	1	68.74 (4.3–1098.99)	34.87 (33.38)	34.87 (3.43)	5.12 (2.49)
Etynodiol*	1	68.74 (4.3–1098.99)	34.87 (33.38)	34.87 (3.43)	5.12 (2.49)
Indoramin	1	68.74 (4.3–1098.99)	34.87 (33.38)	34.87 (3.43)	5.12 (2.49)
Interferon alfa-N1	1	68.74 (4.3–1098.99)	34.87 (33.38)	34.87 (3.43)	5.12 (2.49)
Tandospirone*	1	68.74 (4.3–1098.99)	34.87 (33.38)	34.87 (3.43)	5.12 (2.49)
NEOMYCIN*	1	68.74 (4.3–1098.99)	34.87 (33.38)	34.87 (3.43)	5.12 (2.49)
Tafenoquine	21	55.52 (31.24–98.68)	31.16 (621.93)	31.16 (19.26)	4.96 (4.22)
Finasteride	6	45.83 (16.31–128.75)	27.9 (157.85)	27.89 (11.75)	4.8 (3.49)
Aluminum acetate*	1	34.37 (3.12–379.04)	23.25 (21.6)	23.25 (3.12)	4.54 (2.04)
Corticosteroids	1	34.37 (3.12–379.04)	23.25 (21.6)	23.25 (3.12)	4.54 (2.04)
Difenidol	1	34.37 (3.12–379.04)	23.25 (21.6)	23.25 (3.12)	4.54 (2.04)
Ferric ferrocyanide*	1	34.37 (3.12–379.04)	23.25 (21.6)	23.25 (3.12)	4.54 (2.04)
Levocetirizine	1	34.37 (3.12–379.04)	23.25 (21.6)	23.25 (3.12)	4.54 (2.04)
Clopamide	2	27.49 (5.33–141.72)	19.92 (36.47)	19.92 (5.05)	4.32 (2.36)
Aprotinin*	935	23.8 (22.09–25.64)	17.95 (15128.02)	17.89 (16.81)	4.16 (4.06)
Benzethonium*	1	22.91 (2.38–220.28)	17.43 (15.72)	17.43 (2.62)	4.12 (1.71)
Diphtheria vaccine*	1	22.91 (2.38–220.28)	17.43 (15.72)	17.43 (2.62)	4.12 (1.71)
Mibefradil*	1	22.91 (2.38–220.28)	17.43 (15.72)	17.43 (2.62)	4.12 (1.71)

PRR = proportional reporting ratio, ROR = reporting odds ratio.

* Indicates a new signal not mentioned in the product labeling.

### 3.4. Analysis of inducing events

Analyzing the time to onset of ADRs is of significant importance for drug safety monitoring, clinical medication guidance, regulatory decision-making, and drug development improvements. In this study, the median time to onset of ADRs for Apremilast was found to be the shortest at 17 days. When the shape parameter β of the Weibull distribution is <1, with its 95% CI also <1, the incidence of ADRs is considered to decline over time (early failure curve). Among the top 10 drugs in terms of time to onset before depression events, Varenicline had a median time to onset of depression at 27 days. The time to onset of depression for all 10 drugs belonged to the early failure curve, as shown in Table 5.

**Table 5:** Time to onset and Weibull distribution analysis of adverse drug events related to drug-induced depression.

PT	Time to onset (d)		Weibull distribution analysis		
	Case reports	Median (d)	Scale parameter: α (95% CI)	Shape parameter: β (95% CI)	Type
Varenicline	10,456	27	80.97 (77.61–84.32)	0.68 (0.67–0.70)	Early failure
Isotretinoin	7180	79	296.23 (272.53–319.93)	0.53 (0.52–0.55)	Early failure
Adalimumab	6587	107.5	334.57 (303.84–365.30)	0.65 (0.62–0.68)	Early failure
Oxycodone	6096	152	408.13 (330.66–485.61)	0.64 (0.58–0.71)	Early failure
Interferon beta-1A	5534	285.5	770.91 (720.55–821.27)	0.66 (0.64–0.68)	Early failure
Levonorgestrel	5475	30	213.08 (190.72–235.45)	0.56 (0.53–0.58)	Early failure
Apremilast	4447	17	81.81 (70.89–92.72)	0.61 (0.57–0.64)	Early failure
Duloxetine	4337	47	215.46 (186.94–243.97)	0.55 (0.52–0.58)	Early failure
Natalizumab	3916	144	374.32 (347.12–401.52)	0.67 (0.65–0.70)	Early failure
Oxybate sodium	3492	39	203.46 (145.91–261.01)	0.51 (0.46–0.57)	Early failure

CI = confidence interval.

### 3.5. Sensitivity analysis

**Figure 6.**
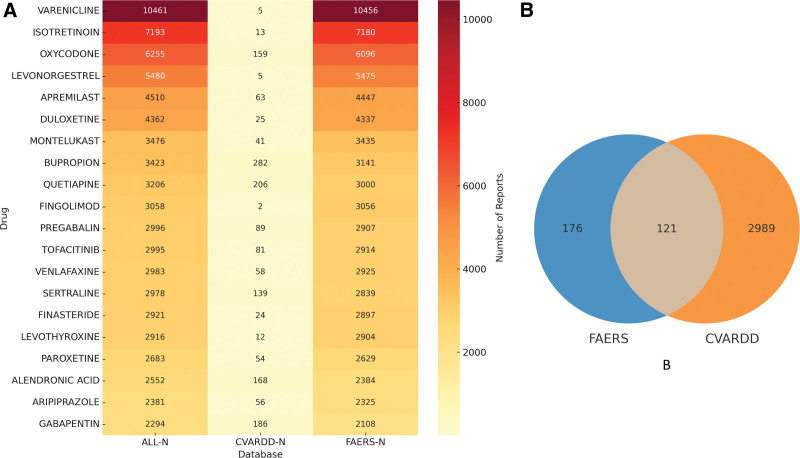
Sensitivity analysis of depression adverse events between FAERS and the Canadian database CVARDD. (A) The top 20 depression risk drugs shared by FAERS and CVARDD. (B) FAERS, CVARDD Depression Risk Medication Heatmap. CVARDD = Canadian Vigilance Adverse Reaction Database, FAERS = Food and Drug Administration Adverse Event Reporting System.

To ensure the robustness of our findings, we further incorporated reports of depression-related AEs from the Canadian Vigilance Adverse Reaction Database (CVARDD) for sensitivity analysis. As shown in Figure 6B, a total of 297 drugs associated with depressive AEs were identified in the FAERS database, whereas the CVARDD database included 3110 such drugs, with 121 overlapping between the 2 databases. This result indicates that although differences exist in the coverage of depression-related drug signals across databases, a certain degree of consistency was also observed, suggesting good cross-database reproducibility and robustness of the signals detected in this study. As shown in Figure 6A, within the overlap between FAERS and CVARDD, 121 drugs were identified, among which the top 20 exhibited strong signal intensities in both datasets. Representative examples include Varenicline, Isotretinoin, Oxycodone, Duloxetine, Bupropion, and Quetiapine, all of which were reported extensively in both FAERS and CVARDD. These findings suggest a high level of consistency in drug signals for depressive AEs across different databases, thereby further strengthening the robustness and reliability of the study conclusions.

## 4. Discussion

In the context of big data, this study innovatively utilized multiple algorithms (ROR, PRR, MGPS, BCPNN) to detect signals of drug-induced depression and visually presented the intersections among algorithms and the ranking of signal intensity through Venn diagrams and forest plots, providing a powerful tool for identifying high-risk drugs.^[26-28]^ Notably, drugs such as APROTININ*, despite numerous reports, had no mention of depression-related AEs in their prescribing information, highlighting the lag in current drug information updates and the necessity of strengthening post-marketing surveillance.^[29-31]^

### 4.1. Epidemiological trends and population characteristics

By conducting an in-depth analysis of AE reports related to depression in the FAERS database up to the third quarter of 2024, this study revealed the epidemiological characteristics, population distribution, and potential risk factors of drug-induced depression globally. Our research not only quantified the number of depression AE reports but also provided a comprehensive perspective on drug-induced depression through time trend analysis, population characteristic portrayal, and drug correlation analysis.

Firstly, in terms of time trends, the number of AE reports with depression as the preferred term has increased annually since the first quarter of 2004, peaking in 2015. This trend coincides with the global increase in antidepressant use and heightened societal attention to mental health. The high coefficient of determination (*R*^2^ = 0.9165) of the polynomial fitting curve indicates that our model accurately captures and explains this long-term growth trend, providing a scientific basis for predicting future occurrences of depression AEs.

Regarding population characteristics, female patients accounted for a larger proportion (61.2%), which may be related to women’s greater susceptibility to mood fluctuations and endocrine changes. Additionally, the age distribution showed a gradual upward trend, with individuals aged 18 to 65 years becoming the primary victims of depression AEs, suggesting that this age group should be the focus of mental health monitoring and intervention.^[32]^ Notably, the highest proportion of individuals weighed between 70 and 90 kg, although the specific mechanism remains to be studied^[33]^; this may be related to differences in lifestyle, metabolic status, or drug response within this weight range.^[34-37]^

### 4.2. Drug analysis and risk signals

In the drug analysis section, we found that drugs such as Varenicline, Isotretinoin, and Adalimumab were most closely associated with depression AEs. Notably, Adalimumab’s prescribing information does not explicitly mention the risk of drug-induced depression, prompting clinicians to enhance monitoring when using it and providing clues for further assessment of drug safety.^[38-41]^ More strikingly, among the top 20 drugs ranked by AE signal intensity, 12 had no mention of depression risk in their labels, revealing numerous potential new AE signals, which are significant for improving the pharmacovigilance system.

### 4.3. Onset time of adverse drug reactions

Furthermore, this study focused on the analysis of ADR onset times, particularly their crucial role in drug safety monitoring, clinical medication guidance, regulatory decision-making, and drug development improvements. Through in-depth analysis of Apremilast and other key drugs such as Varenicline, we not only revealed the specific characteristics of ADR onset times but also further explored their significance in the field of drug safety. Firstly, we found that the median onset time for Apremilast AEs was only 17 days, much shorter than the average onset time for many other drugs, suggesting that Apremilast may produce significant adverse reactions in the short term. This finding has direct implications for clinical medication guidance, prompting doctors to pay special attention to patients’ early reactions when using Apremilast to promptly identify and manage potential adverse reactions.^[42-45]^

Furthermore, we modeled ADR onset times using the Weibull distribution and found that the shape parameter β was <1, with its 95% confidence interval also <1, indicating that the incidence of ADRs declined over time, following the so-called “early failure curve.” This finding is significant for drug safety monitoring and regulatory decision-making. It suggests that for these drugs, early monitoring and intervention may be particularly important, as adverse reactions often concentrate in the early stages of treatment. Therefore, regulatory agencies could require pharmaceutical companies to conduct more rigorous and detailed early safety assessments before drug launch to ensure safety before widespread use. In the analysis of specific drugs, we found that the depression onset times for 10 drugs, including Varenicline, also conformed to the characteristics of the early failure curve, with a median depression event time of 27 days. This result not only further validates the prevalence of the early failure curve but also reveals the high risk of depression, a severe adverse reaction, in specific drugs. For these drugs, both clinicians and patients should remain highly vigilant and closely monitor any abnormal reactions in the early stages of treatment.

### 4.4. Clinical implications and recommendations for drug safety

This study identified significant associations between several widely used drugs and depressive AEs, with some signals not currently reflected in official product labeling. These findings highlight the need for clinicians and pharmacists to strengthen monitoring when prescribing such drugs, particularly in patients who may be vulnerable to psychiatric adverse effects. From a regulatory perspective, updating drug labels to incorporate information on psychiatric risks, including depression, would help enhance clinical vigilance. At the same time, further basic and translational research is warranted to elucidate the underlying mechanisms by which these drugs may trigger depressive symptoms, such as their effects on neurotransmitter systems, endocrine pathways, or other biological processes.

In addition, the advancement of personalized medicine should be emphasized. Tailoring treatment strategies to individual demographic and clinical characteristics may help reduce the likelihood of adverse psychiatric outcomes.^[46-49]^ Multidisciplinary collaboration among psychiatry, internal medicine, pharmacology, and epidemiology experts is also essential to disentangle the complex relationship between pharmacotherapy and depression. Finally, establishing large-scale international cooperative databases and promoting data sharing will further enhance pharmacovigilance capacity, while also supporting public health education by raising awareness of drug-induced depression, encouraging patients and families to recognize early symptoms, and fostering timely reporting of suspected adverse reactions.

### 4.5. Limitation

Despite the strengths of using a large real-world dataset such as FAERS, it is important to acknowledge the inherent limitations of spontaneous reporting systems. First, reporting bias may occur because AEs are reported voluntarily and disproportionately influenced by factors such as media coverage, litigation, or heightened clinical awareness. As a result, the absolute number of reports does not necessarily reflect the true incidence of drug-induced depression. Second, notoriety bias can arise when widely publicized drug-event associations trigger a surge of reports, thereby inflating the perceived risk signal. Third, lag time bias is another concern, as delays between the occurrence of AEs, their recognition, and eventual reporting may obscure temporal patterns and underestimate early risks. To mitigate these biases, we applied standardized data cleaning procedures, removed duplicates, and required positive signals across 4 disproportionality algorithms (ROR, PRR, BCPNN, EBGM) before considering them as valid. Moreover, external validation using an independent pharmacovigilance dataset (CVARDD) helped strengthen the robustness of our findings. Nevertheless, the results should be interpreted as hypothesis-generating signals rather than definitive causal associations, and they warrant further confirmation in pharmacoepidemiological studies and clinical trials.

## 5. Conclusion

In summary, by deeply mining the resources of the FAERS database, this study revealed the epidemiological characteristics, population distribution, and potential risk factors of drug-induced depression, providing a scientific basis and practical guidance for improving the pharmacovigilance system, optimizing clinical decision-making, and formulating public health policies. In the future, we will continue to deepen research in this field and explore more factors influencing drug-induced depression, aiming to provide safer and more effective treatment options for patients.

## Author contributions

**Conceptualization:** Qiuxia Feng.

**Data curation:** Li Yin, Qiuxia Feng.

**Investigation:** Xinping Xiang.

**Methodology:** Xinping Xiang, Li Yin, Chunyan Deng.

**Resources:** Li Yin, Chunyan Deng, Qiuxia Feng.

**Software:** Li Yin, Chunyan Deng, Qiuxia Feng.

**Supervision:** Qiuxia Feng.

**Validation:** Chunyan Deng.

**Visualization:** Xinping Xiang.

**Writing – original draft:** Xinping Xiang, Li Yin, Chunyan Deng, Qiuxia Feng.

**Writing – review & editing:** Xinping Xiang, Qiuxia Feng.
